# Extended longevity of DNA preservation in Levantine Paleolithic sediments, Sefunim Cave, Israel

**DOI:** 10.1038/s41598-022-17399-2

**Published:** 2022-08-25

**Authors:** Viviane Slon, Jamie L. Clark, David E. Friesem, Meir Orbach, Naomi Porat, Matthias Meyer, Andrew W. Kandel, Ron Shimelmitz

**Affiliations:** 1 Department of Evolutionary Genetics, Max Planck Institute for Evolutionary Anthropology, Deutscher Platz 6, 04103 Leipzig, Germany; 2 Department of Anatomy and Anthropology and Department of Human Molecular Genetics and Biochemistry, Sackler Faculty of Medicine, Tel Aviv University, 6997801 Tel Aviv, Israel; 3 The Dan David Center for Human Evolution and Biohistory Research, Tel Aviv University, 6997801 Tel Aviv, Israel; 4 Department of Sociology and Anthropology, George Mason University, MSN 3G5, Fairfax, VA 22030 USA; 5 Institute for Archaeological Sciences, Eberhard Karls University of Tübingen, Hölderlinstr. 12, 72074 Tübingen, Germany; 6 The Role of Culture in Early Expansions of Humans, Heidelberg Academy of Sciences and Humanities at the University of Tübingen, Hölderlinstr. 12, 72074 Tübingen, Germany; 7 The Leon Recanati Institute for Maritime Studies, Department of Maritime Civilizations, School of Archaeology and Maritime Cultures, University of Haifa, Mount Carmel, 3498838 Haifa, Israel; 8 The Haifa Center for Mediterranean History, University of Haifa, Mount Carmel, 3498838 Haifa, Israel; 9 Zinman Institute of Archaeology, School of Archaeology and Maritime Cultures, University of Haifa, Mount Carmel, 3498838 Haifa, Israel; 10 Geological Survey of Israel, 32 Yeshayahu Leibowitz Street, 9691200 Jerusalem, Israel

**Keywords:** Archaeology, Evolutionary genetics

## Abstract

Paleogenomic research can elucidate the evolutionary history of human and faunal populations. Although the Levant is a key land-bridge between Africa and Eurasia, thus far, relatively little ancient DNA data has been generated from this region, since DNA degrades faster in warm climates. As sediments can be a source of ancient DNA, we analyzed 33 sediment samples from different sedimentological contexts in the Paleolithic layers of Sefunim Cave (Israel). Four contained traces of ancient Cervidae and Hyaenidae mitochondrial DNA. Dating by optical luminescence and radiocarbon indicates that the DNA comes from layers between 30,000 and 70,000 years old, surpassing theoretical expectations regarding the longevity of DNA deposited in such a warm environment. Both identified taxa are present in the zooarchaeological record of the site but have since gone extinct from the region, and a geoarchaeological study suggests little movement of the sediments after their deposition, lending further support to our findings. We provide details on the local conditions in the cave, which we hypothesize were particularly conducive to the long-term preservation of DNA—information that will be pertinent for future endeavors aimed at recovering ancient DNA from the Levant and other similarly challenging contexts.

## Introduction

In the last years, paleogenetics studies have been instrumental in reconstructing the histories of past populations, thanks to the successful recovery of DNA from prehistoric humans and animals. The Levant, being a land-bridge between Africa and Eurasia, is of particular interest for conducting such studies, as it has long been recognized as a key region for the migration of humans and animals throughout prehistory^1–5^.

**Figure 1:**
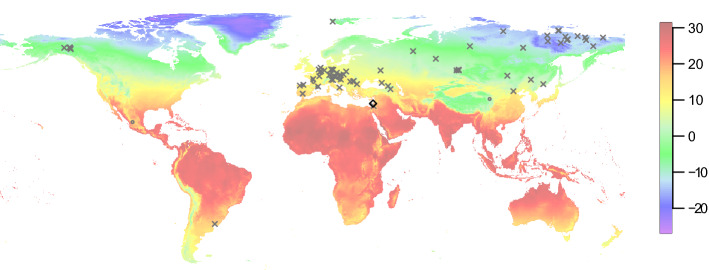
The location of Sefunim Cave compared to other sites where ancient DNA is preserved. The map is colored according to the mean annual temperature (°C), based on climatic data for the years 1970–2000. Sefunim Cave is indicated by the diamond. Other localities from which mammalian DNA from samples dated to at least 30 ka has been retrieved are noted (cross—skeletal remains; circle—sediments). See Supplementary Table S1 for a list of the localities plotted. The map was generated using the ‘maps’ package^87^ in R version 3.5.2 (https://www.r-project.org/).

Warm climates, however, adversely affect the preservation of DNA over time^6–8^, since elevated temperature facilitates degradation processes such as depurination^9^. As a result, ancient DNA studies on specimens found in Pleistocene contexts, particularly prior to the Last Glacial Maximum (LGM, ~ 26.5 to ~ 20 thousand years [ka] ago^10^), have thus far been almost exclusively restricted to regions with temperate or cold climates (Fig. 1, Supplementary Table S1). The oldest DNA recovered to date comes from mammoth remains dated to ~ 1.65 million years ago found in permafrost in Northeastern Siberia^11^. Outside permafrost, DNA up to ~ 400 ka-old has been sequenced from bear and hominin remains in Western Eurasia^12–15^. To date, the oldest DNA retrieved in the Levant originates from human remains dated to between 12 and 14 ka^16^ and the remains of crested rats dated to > 42 ka^17^, providing first genetic glimpses of the end of the Pleistocene. Notably, it has been estimated that when deposited in an environment with a temperature of 20 °C, DNA in bones will break down to an average length of 1 base-pair [bp] after ca. 53,000 years^7^. Although little is known about the long-term survival of DNA in sediments compared to bones, DNA yields from sediment samples have been found to be, at best, similar to yields from skeletal remains originating from the same contexts^18^.

Sediments deposited at archaeological cave sites have recently been shown to be a promising source of ancient hominin DNA, even in the absence of skeletal remains^18,19^. As part of a larger, ongoing study into the characteristics of DNA preservation in archaeological sediments, we collected samples at the site of Sefunim Cave in Israel (Fig. 1)^20,21^ and tested whether they contained traces of ancient mammalian DNA.

**Figure 2:**
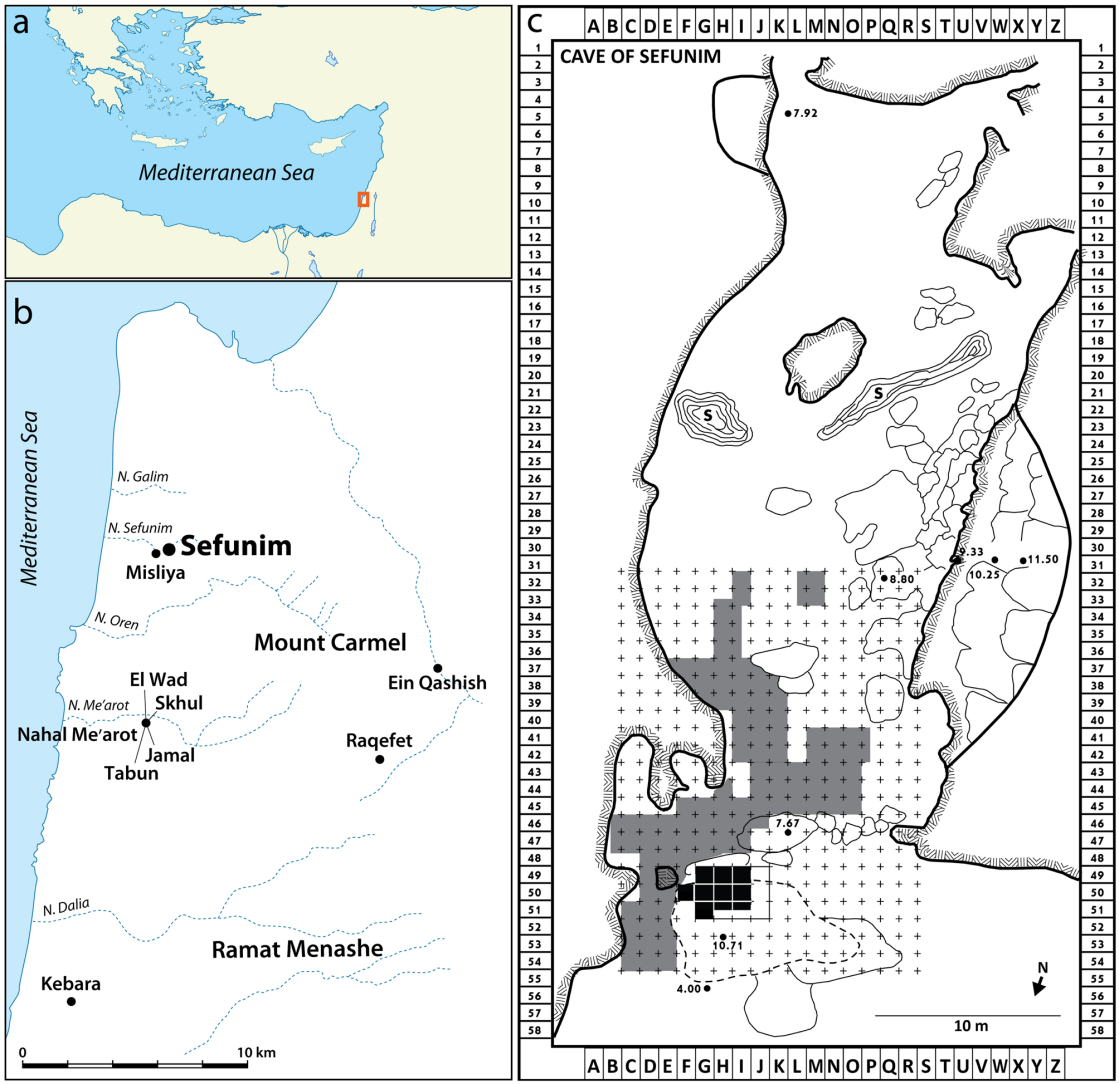
Location maps. (**a**) Map of the Eastern Mediterranean showing the general location of the site; (**b**) map of Mount Carmel with Sefunim and other important sites of the region; (**c**) Plan of Sefunim Cave (after^20^) showing current excavation area (black squares) and Ronen's excavation (grey squares). Maps were generated using Adobe Photoshop and Adobe Illustrator (www.adobe.com).

Sefunim Cave is located in the Mount Carmel on the southern bank of the dry riverbed of Nahal Sefunim (Fig. 2a,b). The site is situated in the Mediterranean climate zone of the Levant, about three kilometers from the current seashore at an elevation of 125 m above sea level. Avraham Ronen, from the University of Haifa, excavated the site extensively during the 1960s, revealing a sequence of layers from the Middle Paleolithic to the historical periods^20^. Renewed excavations at the site since 2013^21^ have focused on the entrance of the cave near the current dripline, an area that Ronen did not previously excavate due to the presence of massive limestone boulders.

**Figure 3:**
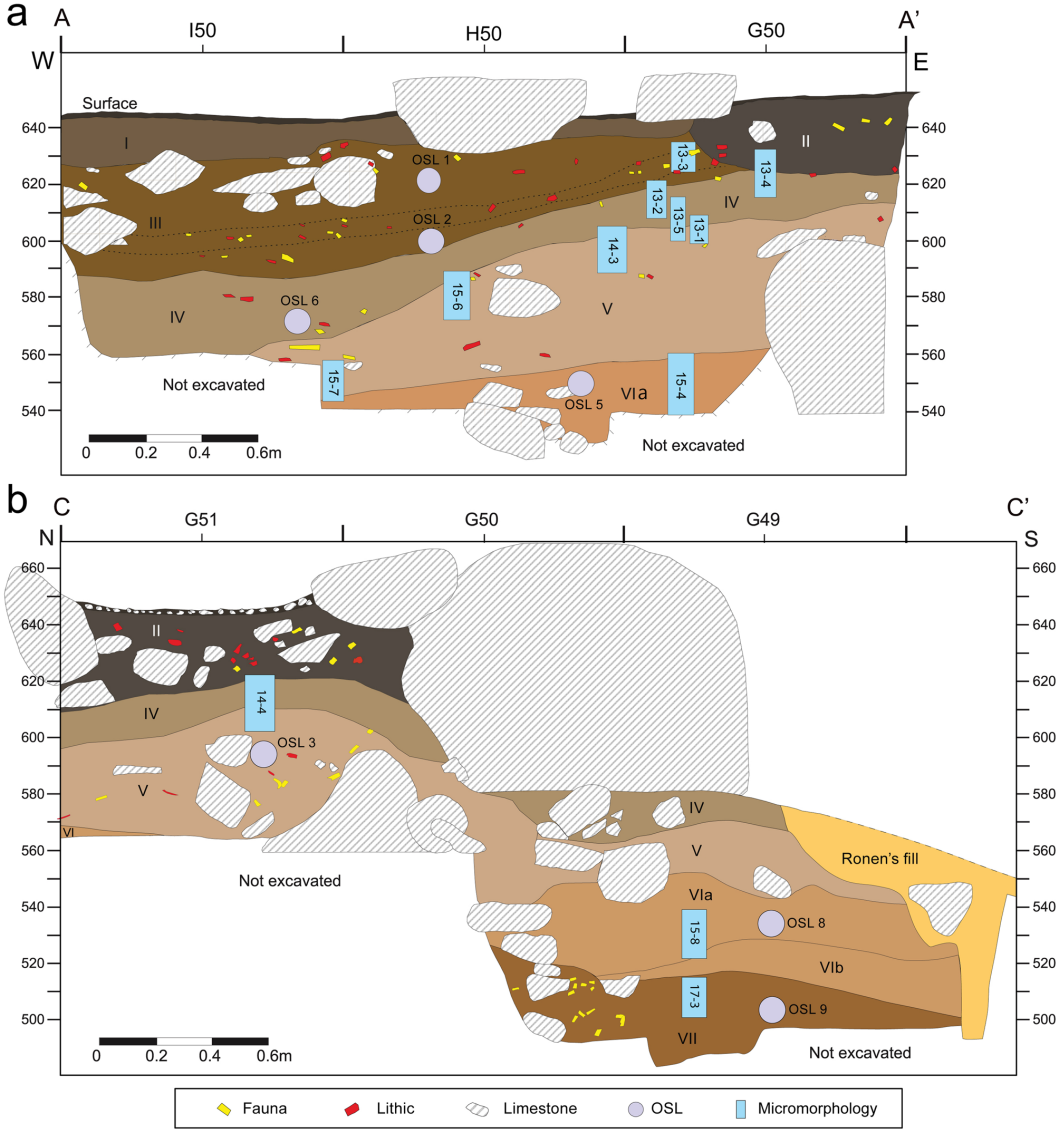
Stratigraphy of Sefunim Cave reflected through the two main sections on the north (**a**) and east (**b**) walls of the excavation. (Sections A–A' and C–C' are depicted in Fig. 4.). Profile drawings were generated using CorelDRAW X7 v.17.5.0.907 (www.coreldraw.com).

**Figure 4:**
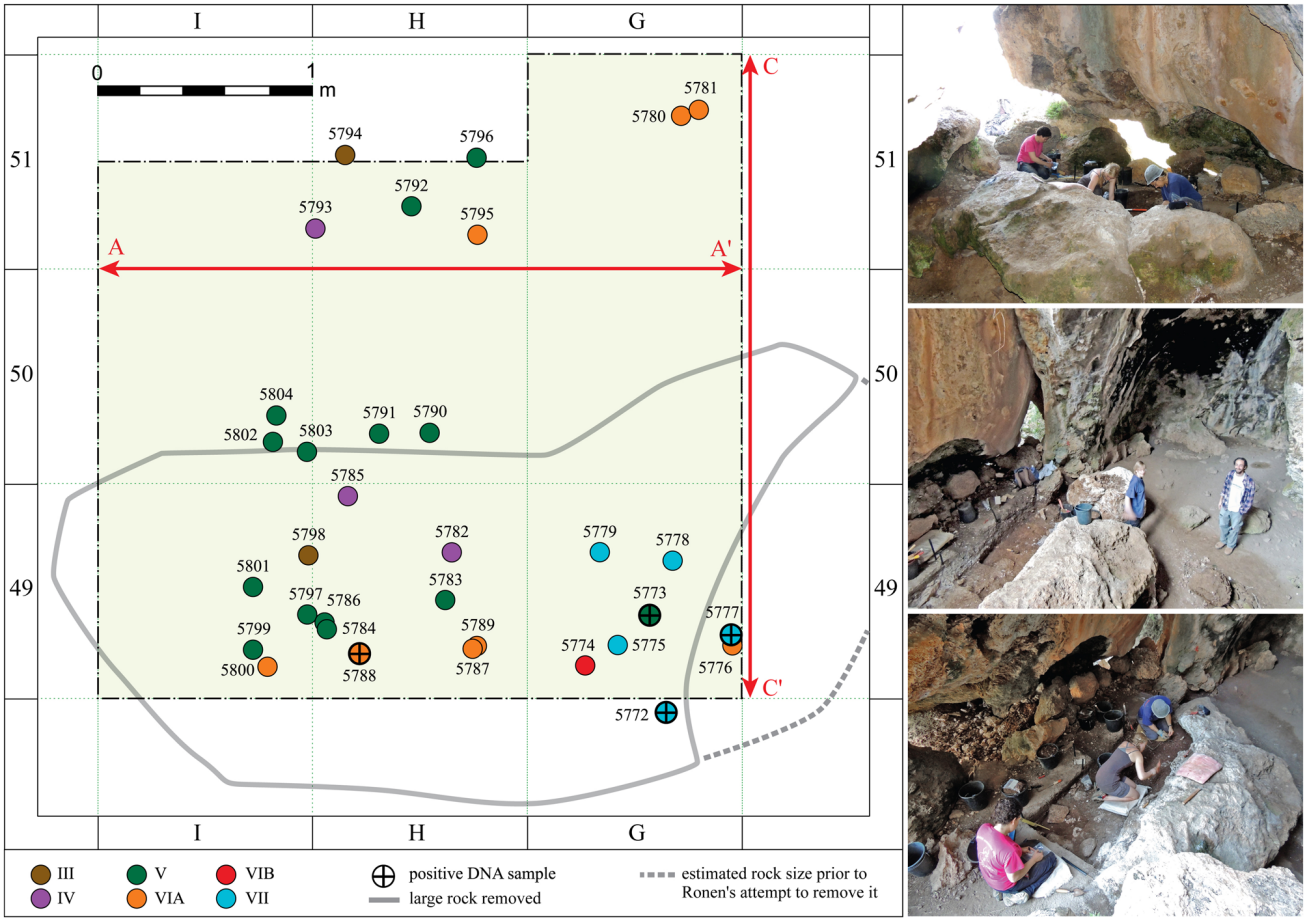
Locations of sediment samples collected for DNA analyses, shown on a plan of the Sefunim Cave excavation. Samples are color-coded according to the layer from which they originate, with a cross indicating samples that tested positive for the preservation of ancient mammalian mtDNA. The gray line depicts the outline of the boulder removed from the excavation area in 2017 prior to sampling. The dashed gray line shows the inferred extent of the rock before Ronen removed part of it. The red arrows represent the two main sections shown in Fig. 3. The photographs on the right document the gradual removal of the limestone boulder before its complete removal. The distribution map was generated with ArcGIS Desktop 10.6.1.9270 (https://desktop.arcgis.com); photographs are by A. Kandel.

These large rocks attest to the gradual collapse of the cave roof (Fig. 2c). While some boulders overlie Holocene deposits, others are embedded within the Late Pleistocene layers (Fig. 3). One of these massive rocks (termed "three drill rock" by Ronen, following his failed attempt to break it apart) was finally removed before the 2017 season. This allowed us to expand the area of excavation (Fig. 4).

The new excavations enabled the identification of five archaeological horizons bearing Paleolithic assemblages. The lowest layer of the sequence (VII) is clearly attributed to the Middle Paleolithic, based on the presence of lithics made using Levallois technology^22^. Overlying this, Layer VI is characterized by a low density of finds and represents deposits between the late Middle Paleolithic (post Layer VII) and the early Upper Paleolithic of Layer V. A minor sedimentological change is noted between the upper and lower parts of layer VI, which were termed VIA and VIB, respectively. Two Emireh points, typical of the Middle to Upper Paleolithic transitional phase in the Levant^23^, are known from earlier excavations at the site. However, we have not yet uncovered such finds within the context of Layer VI. The next horizon is Layer V, containing artifacts assigned to the Levantine Aurignacian complex of the early Upper Paleolithic^24^. This stratum is overlain by Layer IV, attributed to the late Upper Paleolithic, while Layer III is characterized by early Epipaleolithic assemblages^25^. To complete the sequence, Layer II consists of two localized pits which cut into Layers III and IV. While the majority of material in the pits appears to be Epipaleolithic, some of the remains are younger^21^. Layer I includes disturbed material from the surface. The new excavations did not identify any clear Neolithic contexts (unlike Ronen's excavations), with the possible exception of some of the infill in the pits of Layer II.

In this paper we present the results of the screening of sediment samples from Sefunim Cave for the preservation of ancient DNA. We refine the chronology of the site based on new results from Optically Stimulated Luminescence (OSL) and radiocarbon dating. We integrate evidence from the faunal and sedimentological records to further evaluate the depositional and post-depositional processes that contributed to the formation of the site. Finally, we provide details about how we confirmed the *in-situ* status of the Paleolithic sediments and discuss how the depositional environment in one part of the site may have contributed to the preservation of DNA in samples collected there.

## Results

### Retrieval and identification of ancient DNA

A total of 318 loose sediment samples were collected at the site, of which 33 were collected specifically for genetic analyses. These 33 samples covered the five Paleolithic layers III-VII (Fig. 4, Supplementary Data S1). To test whether ancient DNA was preserved in the sediments, we first screened a subset of 11 samples from Layers V, VIA and VII. DNA fragments were extracted from the samples^12,26^ and converted into double-indexed, single-stranded DNA libraries^27,28^, which were then enriched for DNA fragments bearing similarities to mammalian mitochondrial genomes^18,29,30^. After sequencing, the recovered mtDNA fragments were assigned to a taxon of origin at the biological family level^31,32^ and evaluated for the presence of damage indicative of ancient DNA^33^ following the approach in^18^. One library, generated from sample SP5777 from Layer VII, tested positive for the presence of ancient mtDNA fragments. In this library, 15 DNA fragments were assigned to Cervidae. Of these, 100% and 66.7% of fragments starting or ending where the reference genome carries a cytosine (C) (i.e., at the 5′ and 3′ ends, respectively) presented a thymine (T). This constitutes a C to T substitution frequency significantly higher than 10% at both ends, thus exceeding our threshold for defining identified taxa as ancient (Supplementary Data S1).

To further investigate this result and generate more sequencing data, we processed five additional sub-samples from this sample. All five contained traces of Cervidae mtDNA (between 5 and 21 fragments) with elevated C to T changes at their 5′ and/or 3′ ends, although only one of the libraries passed our filtering scheme to be defined as containing ancient DNA (Supplementary Data S1). This confirmed that sample SP5777 indeed retained ancient Cervidae mtDNA, while demonstrating that our power to identify DNA as ancient is limited when low numbers of identifiable fragments are retrieved. For further analyses of this sample, we merged all the fragments assigned to Cervidae from all six libraries (Supplementary Fig. S1).

**Figure 5:**
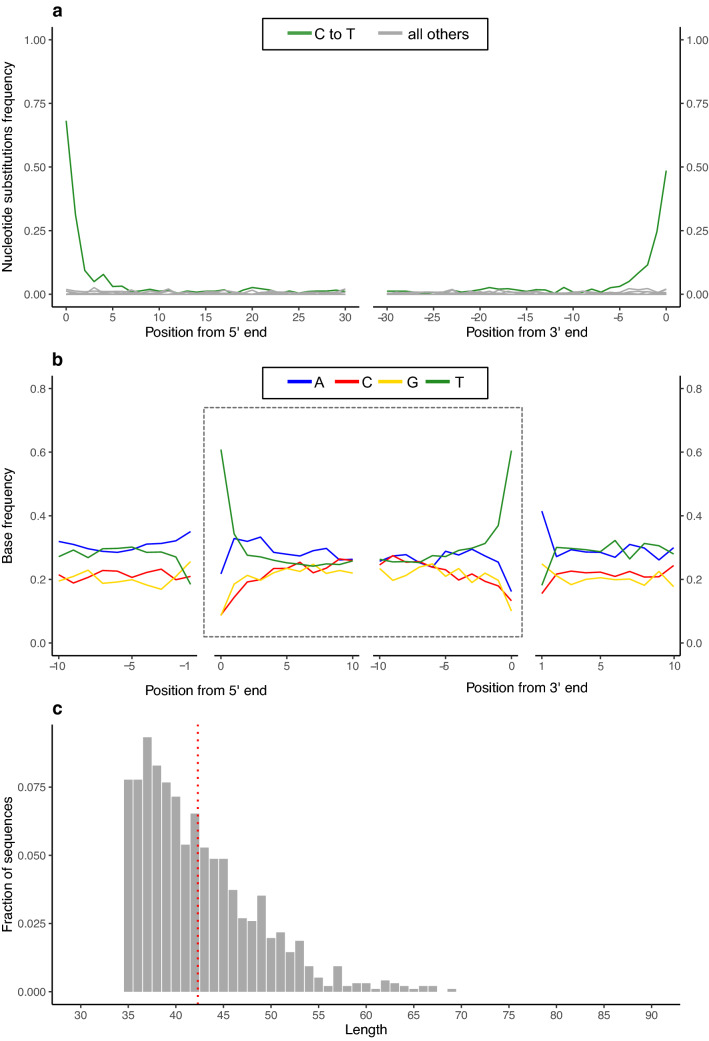
Authentication of the 982 mtDNA fragments assigned to Cervidae in sample SP5773 from Layer V. Following the alignment of the fragments to the *Cervus elaphus* reference mitochondrial genome and their filtering for a mapping quality of 25 or more, 965 fragments were retained for these analyses. (**a**) Frequency of nucleotide substitutions towards the starts and ends of fragments compared to the reference genome. (**b**) Base composition towards the start and end of the sequenced DNA fragments (within dashed gray outline) and of the reference genome upstream and downstream from fragments’ ends. (**c**) Length distribution (in bp) of the Cervidae mtDNA fragments. The average length is shown by the red dashed line.

Motivated by these initial results, we processed the remaining 22 sediment samples, and found that three more preserved ancient DNA fragments (Supplementary Data S1). Sample SP5772, also from Layer VII, contained traces of Cervidae (9 fragments, Supplementary Fig. S1) and Hyaenidae (25 fragments, Supplementary Fig. S1). In sample SP5788 from Layer VIA, we identified 17 fragments attributed to Cervidae (Supplementary Fig. S1). Our richest positive sample was SP5773 from Layer V, in which 982 fragments were identified as originating from Cervidae and 28 fragments from Hyaenidae (Fig. 5, Supplementary Fig. S1). The Cervidae fragments display a 5′ and 3′ C to T substitution frequency of 68.2% and 48.6%, respectively (Fig. 5A). We also note that at the first position upstream and downstream from the ends of the sequenced fragments, the reference genome more often carries an adenine (A) or a guanine (G) than a C or a T (Fig. 5B). This fits the base composition pattern previously observed in ancient DNA recovered from skeletal remains, a pattern which has been interpreted as stemming from depurination resulting in DNA breaks^33^. Lastly, all DNA fragments assigned to Cervidae are shorter than 70 bp, with an average length of 42.3 bp (Fig. 5C). The fact that fragments are short constitutes another feature typical of ancient DNA, as DNA molecules break down over time^7,34,35^.

To test for the presence of DNA from ancient hominins in our samples, we enriched all libraries with probes spanning a human mtDNA genome sequence^36^. However, none of the samples tested positive for the preservation of ancient hominin mtDNA (Supplementary Data S1).

### Phylogenetic inferences using informative positions

To assign the faunal mtDNA fragments to specific species, we used multiple sequence alignments of previously-reconstructed Cervidae or Hyaenidae mtDNA genomes to define “phylogenetically informative” positions within these two families. Similar to the approach in^14^, these were defined as positions in the mtDNA genome where one group of sequences from a genus or species is fixed or nearly-fixed (90% frequency or more) for a base, while all or nearly all other genomes in that family carry another base. We caution that with few DNA fragments to test, and when a large heterogeneity exists in the number of positions available to define the various groups within a family, assignations to groups using this strategy remain tentative.

A preliminary analysis considering twenty groups within the Cervidae family revealed the uncertainty in the determination of positions as informative differences between taxa, among others given the inclusion of taxa that do not belong to the Near Eastern biotope (for details, see Supplementary Materials). To circumvent this issue, we repeated the analysis using a restricted set of informative positions, limited to fixed differences between the four Cervidae groups that are more likely to have been present in the region during the Pleistocene (*Capreolus* sp., *Cervus* sp., *Dama* sp. and *Megaloceros* sp.^37,38^). Between 13 and 231 positions were found to be informative for the differentiation between these four groups (Supplementary Data S2). For the first sample from Layer VII (SP5777), all three fragments overlapping positions indicative of *Cervus* sp. matched the expected base for that group, and no support was found for others. No support was found for any group for the other sample from Layer VII (SP5772, only a single fragment overlapping an informative position); nor for the sample from Layer VIA (SP5788, up to five overlapping fragments). Lastly, all overlapping fragments from the sample from Layer V (SP5773) matched the *Cervus* sp. group (61/61 observations), while none supported any of the other three groups (Supplementary Data S2).

Finally, we used the phylogenetically informative positions defined within Hyaenidae to investigate the mtDNA fragments recovered from samples SP5772 (Layer VII) and SP5773 (Layer V), where we identified this taxon to be of ancient origin. Between 259 and 730 positions in the mtDNA genome were considered to be informative to discriminate between four groups of Hyaenidae (Supplementary Data S2). We find support for the mtDNA fragments coming from spotted hyena (*Crocuta crocuta*) mtDNA (8/8 fragments and 10/10 fragments overlapping informative positions for this group for samples SP5772 and SP5773, respectively), but none for striped hyena (*Hyaena hyaena*), brown hyena (*Parahyena brunnea*) or aardwolf (*Proteles cristata*), based on between 7 and 19 fragments overlapping informative positions for the latter groups (Supplementary Data S2).

### Reconstruction of a partial mtDNA genome

**Figure 6:**
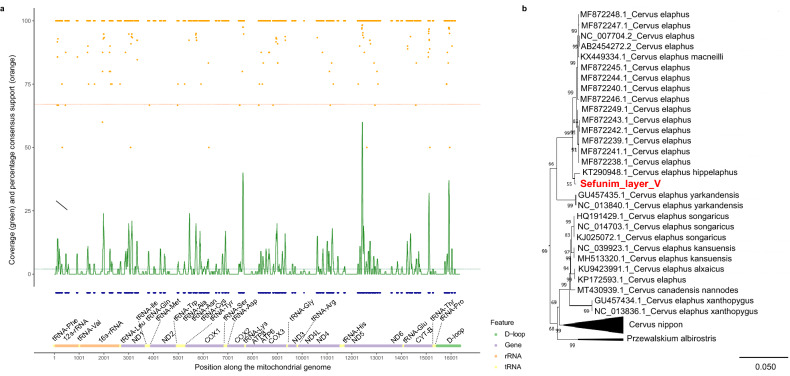
Placing the DNA from Layer V within the Cervidae mtDNA diversity. (**a**) Reconstruction of a partial consensus mtDNA genome sequence, using the alignment to the *Cervus elaphus* reference mitochondrial genome (bottom). Blue dots indicate the positions at which bases could be called (middle). At each position, the coverage (green) and the support for the majority base (orange) is shown (top). The minimum thresholds for these parameters are shown in dashed lines. (**b**) Maximum Likelihood phylogenetic tree based on the partial mtDNA genome sequence reconstructed from sample SP5773 (shown in red) and 380 previously-sequenced Cervidae mtDNA genomes. Branch lengths are scaled based on the number of substitutions per site, and the support for each branch is based on 500 bootstrap replications. Only the part of the tree containing the Sefunim sample is shown, with the full tree presented in Supplementary Fig. S2. The phylogenetic tree was plotted in MEGA X^84^ (https://www.megasoftware.net/).

Using the DNA fragments retrieved from our richest sample, SP5773 from Layer V, we reconstructed a partial cervid mtDNA genome sequence, which may represent a mixture of individuals. We did so by requiring that a given position be covered by at least two fragments, and that at least two-thirds of overlapping fragments carry the same base. This enabled us to call 5392 positions, equivalent to approximately one-third of the mitochondrial genome (Fig. 6A). We note that the probe set we used for targeted enrichment^30^ may not be optimal for recovering complete mtDNA genome sequences, as conserved regions across mammalian species are over-represented.

We then compared the partially-reconstructed mtDNA genome from the sediment sample to 380 previously sequenced mtDNA genomes from extinct and extant Cervidae specimens in a Maximum Likelihood framework. We find that the DNA from the sediment falls within the variation of red deer (*Cervus elaphus* ssp*.*) mitochondrial genomes with high bootstrap support (99% of 500 repeats), although the support for the exact placement within the *Cervus elaphus* clade is relatively low (bootstrap support of 55, Fig. 6B, Supplementary Fig. S2).

### Radiocarbon dating of charcoal and shell

With the recognition of preserved DNA, it became essential to expand the chronology of the site. Based on four previous radiocarbon ages on charcoal, the upper part of Layer V was dated to 30–34 ka calibrated years before present (cal BP)^21^. We submitted a further seven charcoal samples from the main phase of occupation in Layer V and one more from Layer VIA for radiocarbon dating to the Oxford Radiocarbon Acceleration Unit. We also analyzed one dove shell bead (*Columbella rustica*) from Layer V.

**Figure 7:**
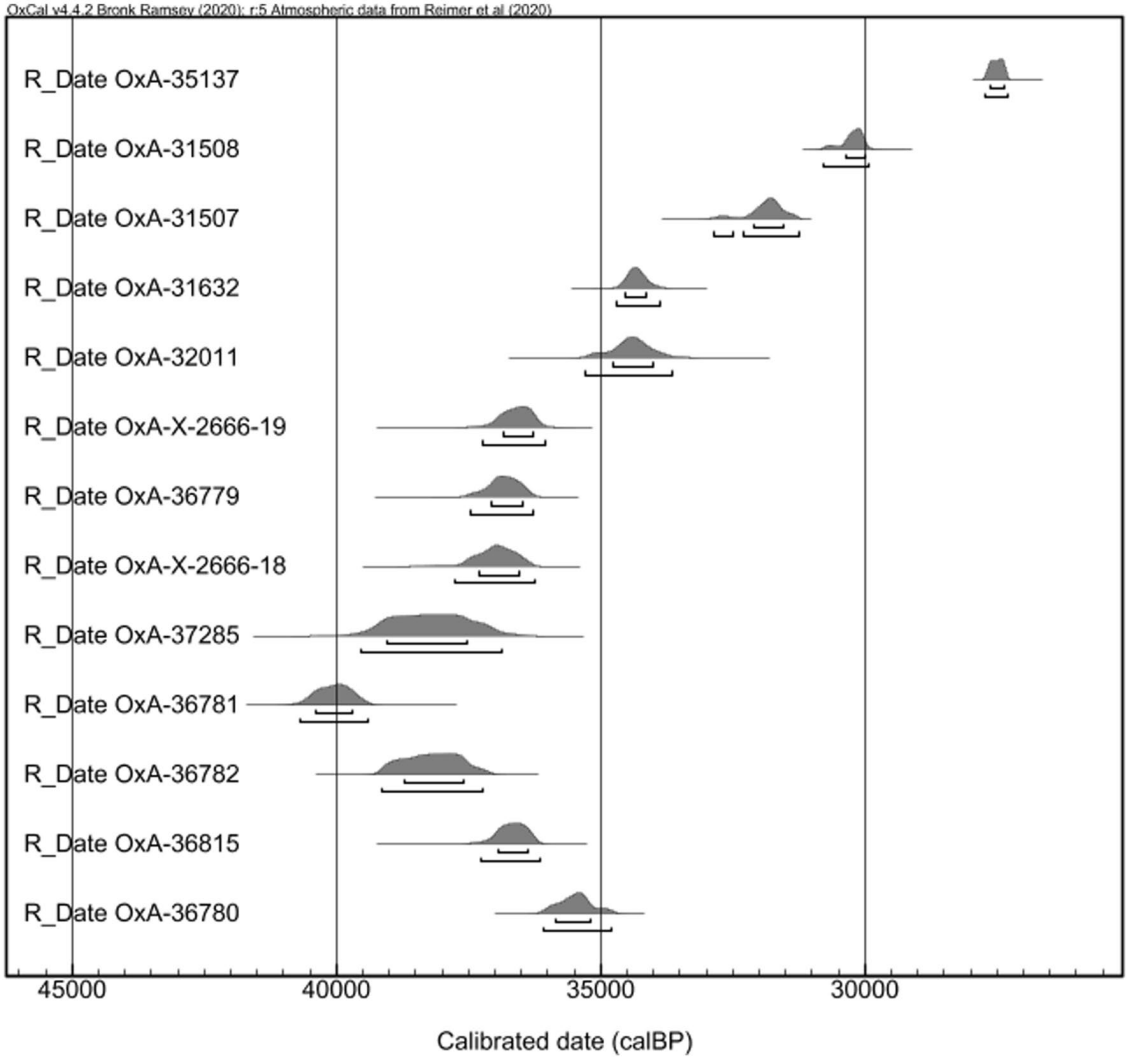
Results of radiocarbon dating in calibrated years before present. Charcoal samples calibrated using IntCal20^39^ and one shell sample (top, OxA-35137) using MarineCal20^41^. Results presented in stratigraphic order from top to bottom, first by layer and then by elevation (below datum). See Supplementary Table S2 for details.

The new results, combined with improvements to the calibration curves^39^, enhance the chronology of the site. Calibration now places the previously dated upper part of Layer V between 30 and 35 ka cal BP, while the underlying main phase of occupation in Layer V dates between 35 and 40 ka cal BP (Supplementary Table S2, Fig. 7). The upper part of Layer VIA also yielded a date of 35–36 ka cal BP, similar to Layer V.

These age determinations of Layer V are consistent with similar occupations in the region. For example, at the nearby Manot Cave^40^, the main Levantine Aurignacian Units 4 and 5 of Area C date between 34 and 38 ka cal BP, while the “Post-Levantine Aurignacian” of Area E dates to 33–34 ka cal BP. Both the chronology and cultural stratigraphy of Manot mirror the results of Sefunim, although the range at Manot is somewhat narrower.

Finally, the shell bead from Layer V yielded a date of 26–27 ka cal BP^41^ without considering the local marine reservoir effect. This date is younger than those from Layers V and VIA. However, the dating of shells can be problematic due to factors such as past variability in the local marine reservoir effect^42^, diagenetic transformation of aragonite to calcite (e.g.^43^), and the difficulty in distinguishing carbon before and after the death of a shell^44^, among others. In fact, some scholars prefer to avoid 14C dating of shells (e.g.^40^). Considering the twelve radiocarbon dates mentioned above, we view the dating of a single shell from Layer V as an anomaly.

### OSL dating of the deposits

Based on previous OSL dating of three samples, we determined the age of Layer III to be 21–24 ka, while Layer V yielded a date of 41 ± 2 ka^21^. To gain further information about the age of the sediments from which ancient DNA was retrieved, we collected six additional samples spanning Layers IV to VII (Table 1, Supplementary Table S3, most sample locations are shown on the sections, Fig. 3).

**Table 1:** Field data, OSL ages and cultural attribution for samples taken.

Lab code	Square	Layer	X	Y	Z	OSL age (ka)	Attribution according to material culture
SEF-1	H51	III	− 7.70	50.50	6.15	20.7 ± 1.1	Early Epipaleolithic
SEF-2	H50	III	− 7.70	49.99	5.97	24.3 ± 1.1	Early Epipaleolithic
SEF-3	G51	V	− 6.30	50.50	5.88	41.2 ± 2.2	Early Upper Paleolithic
SEF-4	H51	V	− 7.24	50.43	5.66	65.3 ± 3.7	Early Upper Paleolithic
SEF-5	H51	VIA	− 7.16	50.02	5.50	66.7 ± 3.8	Early Upper Paleolithic to Middle Paleolithic
SEF-6	I50	IV	− 8.15	49.99	5.72	26.9 ± 1.2	Late Upper Paleolithic
SEF-7	I49	V	− 8.24	48.48	5.59	50.0 ± 3.3	Early Upper Paleolithic
SEF-8	G49	VIA	− 6.10	48.20	5.34	51.0 ± 3.1	Early Upper Paleolithic to Middle Paleolithic
SEF-9	G49	VII	− 6.12	48.20	5.06	71.2 ± 4.1	Middle Paleolithic

Samples SEF-1 to SEF-3 are from ^21^.

All samples show low scatter in equivalent dose (De) values (see ‘OD’ column in Supplementary Table S3) and a roughly normal De distribution. Therefore, the average and error of the De values were calculated using the Central Age Model (CAM;^45^). The laboratory data, dose rates and ages with their associated errors are listed in Supplementary Table S3. The discussion below includes all nine OSL samples from Sefunim. Overall, the De values increase with depth, from 36 to 87 Gy, but the dose rates and carbonate content are not uniform (Supplementary Table S3).

The OSL ages for samples SEF-1, 2 and 6, collected in Layers III and IV, range from 21 to 27 ka and are in accord with the cultural stratigraphy and radiocarbon chronology^25^, representing early Epipaleolithic and late Upper Paleolithic occupations, respectively (Table 1). The samples are typified by relatively high dose rates (1.74–2.26 Gy/ka), generally low De (36–60 Gy), and low carbonate content (6–10%, Supplementary Table S3).

For the three samples from Layer V (SEF-3, 4 and 7), the ages appear to be over-estimated, ranging from 41 to 65 ka. Recall that radiocarbon results from this layer range from 30 to 40 ka cal BP and are consistent with other sites attributed to the Levantine Aurignacian. The De values (45–50 Gy) for the samples from Layer V fall within the range of those from Layers III and IV, but with considerably less variation. However, the dose rates in layer V (0.69–1.11 Gy) are up to a third of the values of layers III and IV (Supplementary Table S3), and carbonate content (48–64%) is up to 10 times higher. If these low dose rates are not representative of the samples’ lifetime and are now lower than during most of their burial, the older calculated ages would be explained. We attribute the low dose rates to pedogenic processes and the mobility of carbonates which were leached from the overlying horizons into Layer V, as implied by the high carbonate content. This process would dilute the clays and reduce the dose rates down to the values measured today.

The deepest three samples (SEF-5, 8 and 9) from Layers VIA and VII yielded OSL ages ranging from 51 to 71 ka (Table 1). While this age is beyond the range of most radiocarbon dating, the Middle Paleolithic lithic technology found in Layer VII supports the OSL ages (the Middle Paleolithic of the Levant is dated between about 250 and 50 ka^46,47^). The case of Layer VIA is more complex, as no clear cultural association currently exists. If we continue to use the dose rate as an indicator of the reliability of the age, then SEF-5 (67 ± 4 ka) might be over-estimated. Like the samples from Layer V, its dose rate is low (0.92 Gy), and its carbonate content is high (50%). While the age falls within the expected range, we cannot be confident in the result based on the scenario described above. On the other hand, samples SEF-8 and 9 appear to be intermediate with regard to dose rate (1.21–1.60 Gy) and carbonate (15–28%). Therefore, we view the ages of 51 ka for Layer VIA and 71 ka for the Middle Paleolithic Layer VII as reliable.

Overall, we found that the results of the OSL dating can be divided into three groups: (1) those that agree with independent radiocarbon dates and estimates of lithic assemblage ages; (2) those that are older than the expected ages; and (3) those for which independent age control was not available. Nonetheless, the results of both OSL and radiocarbon dating are in general harmony with each other and the cultural stratigraphy, despite the over-estimation of OSL results from Layer V and one of the Layer VIA samples. Summarizing the results, we refine the chronology of Sefunim Cave as follows: Layer III dates to 21–24 ka; Layer IV to 27 ± 1 ka; Layer V to 30–40 ka cal BP; Layer VIA to 51 ± 3 ka; and Layer VII to 71 ± 4 ka.

### Comparison with the zooarchaeological record

Next, we compare the taxa identified by genetic analysis to the zooarchaeological record at the site. The most representative sample available so far derives from the dental remains. Since teeth are typically the most diagnostic element, their analysis (including associated cranial and mandibular fragments) provides a general overview of species diversity in the excavated horizons^21^. The results reported here expand upon those presented in^21^. In addition, we see shifts in the assignment of specimens to archaeological horizon as we gained a better understanding of the stratigraphy. The total number of identified specimens (NISP) deriving from the Paleolithic deposits (Layers III–VII) is 1056 (Supplementary Table S4), with zooarchaeological analysis ongoing.

A diverse range of species has been identified at the site. Ungulates dominate the assemblage, with gazelle (*Gazella gazella*) and fallow deer (*Dama mesopotamica*) being the two most commonly identified species in the majority of layers. There is marked variation in the relative frequency of these two species over time. While fallow deer outnumber gazelle in Layers VII and VIA, gazelle dominate the ungulate assemblage in the younger deposits. Cervids are ubiquitous; however, based on our analysis of the dental remains, fallow deer and roe deer (*Capreolus capreolus*) make up the majority of the assemblage. In contrast, only three specimens were identified as potentially deriving from red deer (*Cervus elaphus*): a lower left P3 from Layer VII was confidently identified, while a partially complete right upper M1 or M2 from Layer VII and a right upper dp2 from Layer V could only be identified as cf. *Cervus elaphus*. The zooarchaeological data thus support the DNA-based identification of red deer in Layer V (Fig. 6B, Supplementary Fig. S2, Supplementary Data S2).

We recovered 14 hyena specimens from the layers under consideration; four of these derive from Layer VII, where traces of Hyaenidae mtDNA were identified (Supplementary Table S5;^21^). Most specimens identified thus far are compact elements, such as carpals, tarsals and teeth, which tend to survive better. According to tooth morphology (e.g., the distinct M_1_) and osteological measurements, all of the hyena remains that were identifiable to species level (NISP = 10) belong to the spotted hyena. Only teeth permitted ageing, and the majority of those were juveniles, except for a first molar ascribed to a young adult. It is notable that in Layer VII, three of four teeth belonged to the early juvenile group. Probable hyena coprolites were identified in Layers IV, VIA and VII, although these were too poorly preserved to ascribe to species (for further discussion of the hyena assemblage, see Supplementary Materials).

### Geoarchaeology and site formation processes

Last, we report on a geoarchaeological study of the Paleolithic deposits to contextualize our findings. The results indicate a similar process of sedimentation throughout the archaeological deposits, in which local *terra rossa* accumulated at the site through colluvial deposition. An additional contribution of wind-blown material from the nearby coastal plain is evident by the presence of sub-angular silt to medium-sand sized quartz grains.

Indications of activity are abundant throughout the sedimentary sequence and can be attributed to two main agents: humans and carnivores (probably hyena). It is interesting to note that while evidence for both agents is mixed within all layers, a stratigraphical distinction is observed in their relative proportions (Supplementary Fig. S3). Layers III, V and VII exhibit the highest abundance of microscopic remains associated with human use of the site, including charcoal, burnt clay, burnt bone, wood ash, flint fragments and shell. In contrast, Layers II, IV and VIA contain fewer anthropogenic materials and higher levels of carnivore coprolites, phosphatic grains (probably resulting from the deposition of fecal matter) and unburnt bone. Thus, we interpret the stratigraphic sequence to represent alternating episodes of human and animal activity at the cave.

Post-depositional processes involve minor disturbance to the sediments caused by biological agents (e.g., roots, insects and rodents). This indicates that the deposits and artifacts (mostly large materials such as bones and lithics) are relatively undisturbed and not transported far from their place of initial deposition.

The most important post-depositional process observed at the cave’s entrance involves the dissolution of limestone followed by its precipitation as secondary calcite, a gradual process which cemented the layers differentially. The dissolution of limestone normally results from acidic conditions. Yet we did not identify authigenic minerals associated with elevated acidic conditions. Such minerals are usually very common in Paleolithic caves of the region following the decomposition of bat and bird guano (e.g.,^48–54^). The dissolution of limestone in the upper layers was followed by precipitation of micritic calcite in the lower layers (e.g., Layers V-VII), resulting in the increased cementation of the sediments with depth. Furthermore, layers rich in anthropogenic materials, as observed within the thin section (Layers V and VII), display a higher degree of cementation than the layers associated with higher animal activity (Layers IV and VIA). We attribute this to elevated concentrations of calcitic wood ash resulting from increased anthropogenic activity. Finally, the presence of a three-meter limestone boulder located across the middle of the excavation would likely have acted as a buffer, preventing acidic conditions and promoting better preservation of archaeological materials beneath it.

## Discussion

Here, we present evidence for the preservation of ancient DNA in sediment samples collected in the Paleolithic layers of Sefunim Cave. The DNA fragments we extracted from these sediments present characteristics typical of ancient DNA as established from skeletal remains^33–35^: evidence for cytosine deamination in the form of elevated frequencies of C to T substitutions at the extremities of fragments, a tendency to break at purine sites, and short fragment lengths (Fig. 5, Supplementary Fig. S1). Dating of the relevant layers using radiocarbon and OSL methods suggests that the DNA recovered from the sediment is at least 30,000 years old in the case of Layer V, 51,000 years old in the case of Layer VIA, and as old as 71,000 years in the case of Layer VII. Based on the geoarchaeological study, we see little evidence for the movement of sediments after their deposition, and note that the partial cementation of the sediments increased in depth through layers V to VII.

From most of the sediment samples we tested, relatively few DNA fragments which could be identified as coming from a mammalian mitochondrial genome were recovered (Supplementary Data S1). Thus, in many cases, our power to determine whether the DNA fragments from a given biological family were of genuinely ancient origin—as evaluated based on patterns of nucleotide substitutions—was limited. Indeed, with small numbers, it is possible that a taxon would erroneously appear ancient due to errors or some stochasticity in the appearance of C to T substitutions. On the other hand, it is possible ancient taxa would be missed if the recovered fragments do not pass the thresholds devised to determine authenticity (see “Methods”). Future work to increase the yields of DNA from ancient sedimentary samples would thus be useful, particularly so for studies investigating sites in difficult preservation contexts.

The low number of fragments recovered in some of the samples also limits our resolution to pinpoint the taxa of origin at the species level. Nonetheless, we note that our identification of Cervidae and Hyaenidae (the latter being based on fewer data than the former) matches the faunal assemblage at the site, with the former taxon being represented by bones, teeth and antler, and the latter by bones, teeth and coprolites. Within the Southern Levant, red deer is most commonly identified in deposits dating from the Mousterian to the Natufian^55^. Its presence declines during the Holocene^56^; the latest known occurrence comes from Tell Hesban, where the species was identified in deposits from the twelfth-sixteenth century CE^57^. The spotted hyena thrived during the Middle and Upper Paleolithic in the Southern Levant (^58^: Fig. 1). The last spotted hyenas in the region were found in the Natufian layers (B) in el-Wad and Kebara Caves^59–61^ and in the Neolithic layers in Usba and Sefunim Cave (Layers B and 6–7, respectively^62,63^), but all are from old excavations and the latter two come from poor stratigraphic contexts. The evidence for their presence after the LGM is scarce and they probably went extinct before the Holocene. Nowadays, the spotted hyena is confined to sub-Saharan Africa. The fact that both red deer and spotted hyena are now extinct in Israel further supports that the DNA recovered from the sediments does not originate from present-day fauna.

Our finding of ancient DNA in layers ranging from 30 to 71 ka in the Mediterranean climate of Israel stands out compared to other environments in which mammalian DNA from a similar timeframe or older have been recovered (Fig. 1), and provides the first evidence for the preservation of ancient DNA in Pleistocene sediments from warm climate. We note that all four positive samples were taken from the same area within the cave (Fig. 4, Supplementary Data S1), suggesting that local conditions were particularly conducive to the preservation of DNA over time. In fact, the samples were collected beneath or adjacent to the location where we removed a three-meter long limestone boulder at the start of the 2017 field season (Fig. 4) (all aDNA and six OSL samples were collected thereafter). The boulder lay on the early Epipaleolithic layers, suggesting a collapse about 21–24 ka ago. Note that the estimated date of collapse of the boulder constitutes an additional minimal age constraint for the DNA we recovered in the sediments beneath it.

Several aspects may have contributed to the preservation of ancient DNA at Sefunim Cave. While the exact impact of many micro-environmental factors that may affect DNA preservation in sediments (e.g., mineral content, pH)^8,64^ remains undetermined, it has been shown that DNA decays faster in unstable environments (e.g., in contexts with high thermal fluctuations)^8^. Indeed, it has been suggested that the formation of ‘closed’ or ‘semi-closed’ systems in other substrates contributes to the longevity of DNA preservation. For instance, the consistently higher rates of DNA preservation in the petrous part of the temporal bone compared to other skeletal elements has been attributed to the higher bone density of the former, which may protect the DNA from *post-mortem* damage due to either bacterial or chemical processes^65^. Likewise, the fact that stalagmites form closed systems has been inferred to drive the survival of DNA in them over extended timeframes^66^. Sefunim Cave constitutes a generally closed cavity, characterized by cooler and more stable temperatures than its surroundings^20^, and it is located on the north-facing slope of the wadi, which is characterized by relatively stable conditions compared to the south-facing one^67^. Yet, within the Mediterranean landscape, we hypothesize that the major contributor to the long-term preservation of DNA at the site is the above-mentioned large boulder that protected the sediments underlying it, reducing the loss of DNA from them over time. We speculate that the boulder falling on top of the sediments acted to form a relatively stable, ‘semi-closed’ micro-environment, thereby lessening the degradation of DNA in that part of the site compared to others. In particular, the massive limestone rock may have contributed to a more stable temperature underneath it; may have protected the underlying sediments from humidity, rainfall or surface runoff percolating through the site near its entrance; and its calcium carbonate content may have locally buffered the acidity in the underlying soil.

Because sediment is ubiquitous and readily-available at archaeological sites, it is a useful material for testing ancient DNA preservation. However, even when coupled with targeted enrichment methods, DNA extracted from sediments often represents a mixture of taxa and tends to be recoverable only in low amounts^18,68^. We have established that ancient DNA is preserved at Sefunim Cave and determined areas within the excavation where such fragments are more likely to be recovered. This information will guide future sampling efforts for DNA work at the site, and may also have applications at other sites in the region, where one hopes to retrieve ancient DNA from sediments and/or skeletal remains. The presence of ancient DNA in Pleistocene sediments from the Levant also increases the odds of recovering older human DNA from the region by focusing screening efforts on sites and excavation areas showing the most promising levels of mammalian DNA preservation, irrespective of whether they yielded human skeletal remains. Such screening efforts could target the sediments directly or use collagen peptide mass fingerprinting^69^ to identify human remains among undiagnostic bone fragments. The recovery of human DNA would make it possible to conduct genetic analyses on the populations that created the rich Paleolithic record of the Levant, filling in a gap in the current paleogenetic record.

To summarize, the DNA obtained from sediment at Sefunim Cave displays an extended longevity compared to theoretical expectations of DNA survival in warm climates^7^. Our results demonstrate that under the right circumstances, the retrieval of DNA from Upper and Middle Paleolithic contexts is feasible, even in sediments, in regions where conditions of preservation are presently considered unfavorable.

## Materials and methods

### Ancient DNA analysis

#### Generation of sequencing data

Thirty-three sediment samples were taken at the site of Sefunim Cave, Israel, during the 2017 excavation season specifically for DNA analysis. To minimize the introduction of contamination by present-day human DNA, the collectors wore gloves and a face mask, changing their sampling and protective equipment after each sample. Furthermore, they collected samples in sterile tubes using disposable scalpels after the removal of 1–2 mm of surface material.

The samples were processed in the ancient DNA laboratory at the Max Planck Institute for Evolutionary Anthropology (Leipzig, Germany) in three sets (Supplementary Data S1). The first, constituting a preliminary screening for DNA preservation in the sediment from the site, was comprised of 11 samples collected from layers V, VIA and VII. The second set consisted of five additional sub-samples from sample SP5777, which had tested positive in the preliminary screening. The third set included the remaining 22 samples, spanning all five Paleolithic layers at the site.

For DNA extraction, between 41.5 and 80.5 mg of sediment were sub-sampled using disposable anti-static plastic spatulas. DNA was extracted using a silica-based method, performed either manually as described in^12^ with the modifications in^70^; or on an automated liquid handling platform (Bravo NGS workstation, Agilent Technologies) with binding buffer ‘D’ as described in^26^.

DNA fragments were converted into DNA libraries using a single-stranded library preparation method^71^ implemented on the automated liquid handling platform^28^. A short artificial oligonucleotide was spiked into each library in order to assess whether inhibitory substances potentially co-extracted with the DNA interfered with the library preparation^72^. The number of spike-in oligonucleotides and the overall number of DNA molecules comprised in each library were estimated by qPCR assays^28,72^. Each library was then tagged with two unique indexes^27^ and amplified to PCR plateau.

An aliquot of each indexed library was enriched for mitochondrial (mt) DNA fragments using two sets of probes: one encompassing the mtDNA genome sequence of 242 mammals^30^, to test for the presence of ancient DNA of any mammal in the library; and the other based on the reverse Cambridge Reference Sequence (rCRS) of the human mtDNA genome as described in^36^, to test specifically for the preservation of ancient hominin mtDNA. Targeted enrichment was performed on the automated liquid handling platform using either an on-beads hybridization capture protocol^29^ with the modifications in^18^; or using an in-solution protocol^36^.

A list of the extracts and DNA libraries generated from each sample and from the negative controls is shown in Supplementary Data S1.

Libraries from each targeted enrichment scheme were pooled (along with libraries generated for other projects), and paired-end sequencing in 76 cycles was performed on an Illumina MiSeq platform. After sequencing, bases were called using Bustard^73^. Adapter sequences were removed and full-length sequences were reconstructed by merging overlapping forward and reverse reads using leeHom^74^. For all downstream analyses, only sequences carrying the exact expected double-index combination for each given library were considered.

#### Identifying ancient DNA in the mammalian mtDNA capture data

Sequenced DNA fragments were attributed to a taxon of origin following the strategy described in Ref.^18^. Briefly, fragments were aligned to the concatenated mtDNA genome sequences of the 242 mammalian taxa comprised in the probe set using BWA^75^ with parameters suited for ancient DNA^76^. Using SAMtools^77^, we filtered for a minimum sequence length of 35 base-pairs (bp), as this length cutoff was the one tested and validated for our analytical pipeline using simulated data in^18^. To filter out PCR duplicates while minimizing the effect of sequencing errors, exact sequence duplicates were removed, but only sequenced DNA fragments seen at least twice were retained. These were then compared to a non-redundant database of reference mammalian mitochondrial genomes (RefSeq database at the National Center for Biotechnology Information [NCBI]^78^) using BLAST^31^, the output of which was parsed using the “lowest common ancestor” algorithm implemented in MEGAN^32^. Taxonomic assignments were retained at the biological family level, with a family considered to be present in the data if at least three DNA fragments and at least 1% of identifiable fragments were assigned to it.

The DNA fragments assigned to each family were evaluated for the presence of damage indicative of ancientness, i.e., the replacement of cytosine (C) bases by thymine (T) bases at the ends of fragments due to the deamination of cytosines to uracils over time^33^. To do so, fragments were re-aligned using BWA as above to a reference mitochondrial genome representative of that family (see Supplementary Data S1), and filtered for a mapping quality of at least 25. To consider a family to be of ancient origin, we required the DNA fragments to carry significantly more than 10% C to T substitutions to the reference genome on both their first (5′) and last (3′) positions^18^, as tested using a one-tailed binomial test with hypothesized probability of success = 0.1, as calculated using the ‘binom.test’ function in R^79^. For the plot shown in Fig. S1E relevant to sample SP5777, we merged all fragments assigned to Cervidae from all six libraries with SAMtools^77^.

Additionally, we noted the base composition of the reference genome upstream and downstream of the sequenced DNA fragments as well as within the fragments themselves using MapDamage 2.2.0^80^. Lastly, we computed the mean and the distribution of fragments’ lengths in R^79^.

#### Phylogenetic analyses

To further elucidate the taxon of origin for DNA sequences in datasets deemed to be ancient, we generated sets of phylogenetically informative positions in the Cervidae and Hyaenidae mtDNA genomes. To do so, we downloaded from the Nucleotide database at NCBI^81^ all complete or nearly-complete mtDNA genome sequences pertaining to these two families, filtering for genome sequence length between 12,000 and 17,000 bp for Cervidae and between 12,000 and 20,000 bp for Hyaenidae. This resulted in 378 mtDNA genomes pertaining to Cervidae (downloaded May 2020), to which the two ancient *Megaloceros giganteus* mtDNA genome sequences from^82^ were added. For the Hyaenidae, 48 mitochondrial genome sequences were compiled (downloaded October 2020). Multiple sequence alignments were generated, with the *Cervus elaphus* (NC_007704.2) and *Crocuta crocuta* (NC_020670.1) mtDNA genomes used as references for alignment using MAFFT with 1000 cycles of iterative refinement^83^. Following the scheme in^14^, within each family, we then searched for positions where all mtDNA genomes pertaining to a given group carry an identical base, which differs from all other genomes in that family. Groups were defined based on the genus indicated in the sequence name. This resulted in a total of 1691 informative positions for Hyaenidae (Supplementary Data S2). For the Cervidae, given the variety and the large number of taxa included, as well as the heterogeneity in the number of genomes from each group in the NCBI database, no fixed differences were found to enable the identification of ten groups. For these, we relaxed the criterion to define informative positions, requiring that at least 90% of genomes pertaining to the given test group carry an identical base, which had to differ from 90% of all others. This allowed us to generate a list of informative positions for nine groups (*Alces alces* spp., *Axis* sp., *Cervus* sp., *Dama* sp., *Mazama* sp., *Odocoileus* sp., *Pudu* sp., *Rucervus* sp. and *Rusa* sp.). No phylogenetically informative positions could be determined for the *Przewalskium albirostris* group. Overall, a total of 704 informative positions were retained for Cervidae (Supplementary Data S2). A second set of informative positions for Cervidae was generated by retaining only mtDNA genome sequences pertaining to groups that are known to have existed in the Levant during the Pleistocene. Fixed differences between these four groups (*Capreolus* sp., *Cervus* sp., *Dama* sp. and *Megaloceros*) were required to define the positions, yielding a set of 311 informative sites (Supplementary Data S2). At informative positions, we noted the base carried by the DNA fragments in our datasets (aligned to the above-mentioned reference genomes to maintain the same coordinate system), while masking T bases on forward and A bases on reverse strands within the three first and three last alignment positions to dampen the effect of cytosine deamination at the ends of fragments on our analysis.

Using the sequencing data from sample SP5773 aligned to the *Cervus elaphus* reference genome, we called a partial consensus mtDNA genome sequence by a majority vote. To call a base, we required the position to be covered by at least two fragments and that at least 67% of fragments carry the same base. As above, to avoid spurious results due to cytosine deamination, we masked T bases and A bases on fragments sequenced in the forward and reverse orientations, respectively, at the first three and last three alignment positions.

The reconstructed mtDNA genome sequence from sample SP5773 was aligned to the above-mentioned 380 Cervidae mtDNA genomes using MAFFT^83^. A phylogenetic tree was reconstructed using the Maximum Likelihood method implemented in MEGA X^84^. The substitution model was chosen based on having the lowest Bayesian Information Criterion (BIC) score. The Hasegawa-Kishino-Yano model^85^ was used, with a discrete Gamma distribution of evolutionary rate differences among sites and allowing some sites to be evolutionarily invariable (HKY + G + I model). Fewer than 5% alignment gaps, missing data, and ambiguous bases were allowed at any position (“partial deletion” option). The support for each branch was evaluated by performing 500 bootstrap repetitions.

#### Analysis of the human mtDNA capture data

The processing of the sequencing data generated following the enrichment of DNA libraries with probes based on the human mtDNA genome was similar to the processing of data after enrichment for mammalian mtDNA fragments, with the following exceptions: first, the data was aligned to the rCRS and filtered for length of at least 35 bp and mapping quality of at least 25; and second, PCR duplicates were removed by collapsing replicate sequences based on their alignment positions using bam-rmdup (https://github.com/mpieva/biohazard-tools/). Taxonomic assignment using BLAST and MEGAN was carried out as described above. The alignment to the rCRS was used to compute the frequencies of C to T substitutions at the ends of the sequenced DNA fragments.

#### Plotting of comparative data

The world-wide annual mean temperature shown in Fig. 1 is based on climatic data collected for the years 1970–2000 downloaded from the WorldClim database at 10-min resolution (equivalent to 344 km^2^ at the equator)^86^. To show sites where the preservation of mammalian DNA in samples dated to at least 30 ka has been previously demonstrated, we searched the literature for studies on ancient skeletal remains and/or sediments, where the sequencing data was generated using DNA library preparation methods, allowing for the evaluation of DNA damage patterns in the sequenced fragments. See Supplementary Table S1 for the list of localities plotted.

### OSL dating

Six samples for OSL dating were collected in August 2017 from Layers IV to VII (Supplementary Table S3), in addition to the three samples already taken during the 2014 season^21^. Complementary sediment samples were collected from the same locations for dose rate evaluations.

Sample preparation and quartz extraction were the same as in Ref.^21^. Equivalent dose values (De) of the purified quartz were measured using the single aliquot regenerative (SAR) dose protocol on 19 2-mm aliquots. Previous dose recovery results indicated that the most suitable measurement conditions are a preheat of 10 s at 260 °C, a test dose of ~ 9 Gy and a test dose preheat of 5 s at 200 °C^21^; these were used for all samples.

Alpha, beta and gamma dose rates were evaluated from the radioactive elements measured on the complementary sediment sample by inductively coupled plasma mass spectrometry (U and Th) or inductively coupled plasma atomic emission spectroscopy (K). The cosmic dose rate was estimated from a burial depth of 5 m (to include the cap rock) and moisture content was estimated at 20 ± 4% for all samples, taking into account the clayey nature of the sediment, the local Mediterranean climate and seasonal variations.

### Zooarchaeological analysis

The zooarchaeological analysis is ongoing; in order to gain a basic understanding of species diversity, we prioritized the analysis of dental remains and the associated cranial and mandibular fragments. Given that teeth are readily identifiable as such, they were separated during the initial sorting of the bucket finds. This means that we were able to incorporate both piece-plotted specimens and those from the bucket finds; as such, the dental remains provide the most representative sample available to date. Analysis took place in 2015 and 2017, and taxonomic identifications were made by JLC using the comparative collections housed at the University of Haifa (Laboratory of Archaeozoology) and at the University of Tübingen (Institute for Archaeological Sciences). Specimens identified as hyenid were submitted to MO for further analysis; taxonomic identification of these specimens took place in 2019 using the comparative and fossil collections in Tel Aviv University and the British Museum of Natural History in London.

### Geoarchaeology and site formation processes

Geoarchaeological study of the Paleolithic deposits revealed by the renewed excavations at Sefunim Cave included the collection of 26 undisturbed monolithic sediment blocks covering the entire sedimentary sequence attributed in the field to Layers II to VII^21^. In total, 43 thin sections were produced at the Geoarchaeology Laboratory at the University of Tübingen (Germany), measuring 60 × 90 mm. The thin sections were studied under the naked eye and under magnification (25X–200X) using a Zeiss Axio Imager petrographic microscope with plane-polarized light (PPL), cross-polarized light (XPL) and blue-light fluorescence. Each thin section was qualitatively assessed in order to evaluate the relative abundance of: (1) fire residues, including rubified clay aggregates, micro-charcoal, wood ash pseudomorphs and burnt bones; (2) micro-flint (<2 mm); (3) shell fragments (marine and terrestrial); (4) phosphatic grains; (5) micro-bones (< 2 mm); and (6) the extent of calcitic cementation of the matrix.

### Informed consent

The authors state that informed consent for publication of identifying information/images was obtained.

## Supplementary Information

Supplementary Information 1.

Supplementary Information 2.

Supplementary Information 3.

## Data Availability

Sequencing data generated for this project have been deposited in the European Nucleic Archive (ENA), with accession ID PRJEB44172 (https://www.ebi.ac.uk/ena/browser/view/PRJEB44172).
