# Supplementary material for: Extended longevity of DNA preservation in Levantine Paleolithic sediments, Sefunim Cave, Israel
Source: Sci Rep. 2022 Aug 25;12:14528. doi: 10.1038/s41598-022-17399-2 (PMC9411205; doi:10.1038/s41598-022-17399-2)
Supplement: Supplementary file 3 — Supplementary Information 3. [file 41598_2022_17399_MOESM3_ESM.pdf]

SUPPLEMENTARY MATERIALS

Extended longevity of DNA preservation in Levantine Paleolithic sediments, Sefunim Cave, Israel

Viviane Slon[1,2,3,*], Jamie L. Clark[4,5,6], David E. Friesem[7,8], Meir Orbach[9], Naomi Porat[10], Matthias Meyer[1], Andrew W. Kandel[6], Ron Shimelmitz[9]

1 – Department of Evolutionary Genetics, Max Planck Institute for Evolutionary Anthropology, Deutscher Platz 6, 04103 Leipzig, Germany

2 – Department of Anatomy and Anthropology and Department of Human Molecular Genetics and Biochemistry, Sackler Faculty of Medicine, Tel Aviv University, Tel Aviv 6997801, Israel

3 – The Dan David Center for Human Evolution and Biohistory Research, Tel Aviv University, Tel Aviv 6997801, Israel

4 – Department of Sociology and Anthropology, George Mason University, MSN 3G5 Fairfax, Virginia 22030, USA

5 – Institute for Archaeological Sciences, Eberhard Karls University of Tübingen, Hölderlinstr. 12, 72074 Tübingen, Germany

6 – The Role of Culture in Early Expansions of Humans, Heidelberg Academy of Sciences and Humanities at the University of Tübingen, Hölderlinstr. 12, 72074 Tübingen, Germany

7 - The Leon Recanati Institute for Maritime Studies, Department of Maritime Civilizations, School of Archaeology and Maritime Cultures, University of Haifa, Mount Carmel, 3498838, Haifa, Israel

8 - The Haifa Center for Mediterranean History, University of Haifa, Mount Carmel, 3498838, Haifa, Israel

9 - Zinman Institute of Archaeology, University of Haifa, Mount Carmel, 3498838, Haifa, Israel

10 – Geological Survey of Israel, 32 Yeshayahu Leibowitz Street, Jerusalem 9691200, Israel

* Correspondence to viviane@tauex.tau.ac.il (VS).

Supplementary text

Preliminary analysis of Cervidae mtDNA fragments

In order to assign the Cervidae mtDNA fragments we recovered at a species or genus level, we put together a list of positions in the mitochondrial genome that would be informative to differentiate between sequences grouped by species or genus, based on a multiple sequence alignment of 380 Cervidae mitochondrial genome sequences. Between one and 291 positions were determined to discriminate each of the twenty groups we considered (Supplementary Data S2). We then examined the state carried by fragments attributed to this family in all four positive samples at the aforementioned informative positions. One sample from Layer VII (SP5777) had up to 33 fragments overlapping group-defining informative positions, while the other (SP5772) had three such fragments. None of these showed support for any of the 20 groups tested. For the sample from Layer VIA (SP5788), the single fragment overlapping a defining position for the fallow deer (*Dama* sp.) showed support for that group, while others for which overlapping fragments were recovered were not supported (based on between 0/1 and 0/7 fragments). As for the sample from Layer V (SP5773), the single fragment overlapping one of the four positions informative for determining an attribution to *Cervus* sp. matched the expected base for that group. Low support was found for the Pudú (*Pudu* sp.) group (7.4% of overlapping fragments, 6/81 observations) and the *Rusa* sp. group (18.5% of fragments, 5/27 observations). However, in both cases, the support for the tested group comes from fragments overlapping a single position, possibly reflecting the uncertainty in the determination of these positions as informative differences between groups. This is further emphasized by the fact that these two groups do not belong to the Near Eastern biotope, being rather South American and South Asian genera, respectively. Little to no support (up to 2.3% of fragments) was found for the other groups tested (Supplementary Data S2). Additional analyses were carried out after restricting to taxa likely to have been present in the region during the timeframe in question, as detailed in the main text.

Hyenas at Sefunim Cave

The spotted hyena is the most abundant carnivore in the Paleolithic faunal record of the Southern Levant[58], except for fox, which was probably incorporated as part of human resource exploitation[88]. Hyena remains together with evidence of their daily life (e.g. gnawed and digested bones, coprolites) were found in almost every Paleolithic cave site of the Southern Levant[58,89]. Humans and hyenas likely used the caves in alternation. Dissecting the stratigraphic relationships of these alternating human and hyena occupations is thus crucial to Paleolithic archaeology[90]. In addition, since humans and hyenas lived in the same environments, subsistence preferences of hyena can serve as an external reference for contemporaneous human hunting activities. Furthermore, hyenas might have competed with humans for both resources and living places[58]. The role played by humans and hyenas in accumulating the deposits at Sefunim Cave is a focal point of ongoing research and will be addressed in future publications on the geoarchaeological and zooarchaeological records.

Hyenas, like humans, are considered to have dispersed out of Africa into the rest of the world[5,91]. However, hyena populations vary geographically, with body size decreasing with latitude population in accordance with Bergman's rule[60]. The European cave hyena is larger than the Levantine population, which in turn is larger than the recent African population. While most teeth of the Levantine form are smaller on average, the canines are as large as in European cave hyena. Thus, there is also a relative size difference. The Levantine spotted hyena differs from the cave hyena in having relatively more powerful canines and weaker molars, pointing to modifications in diet. Remarkably, the molecular research shows that those hyena populations belongs to one species, *Crocuta crocuta*[5,91]. Although the Levant may have been a corridor for dispersal events of hyenas, no genetic data exists from this region to date. Thus, reconstructing the population history of hyenids and their ethology is of great importance to human evolution and paleoanthropology research.

Supplementary figures

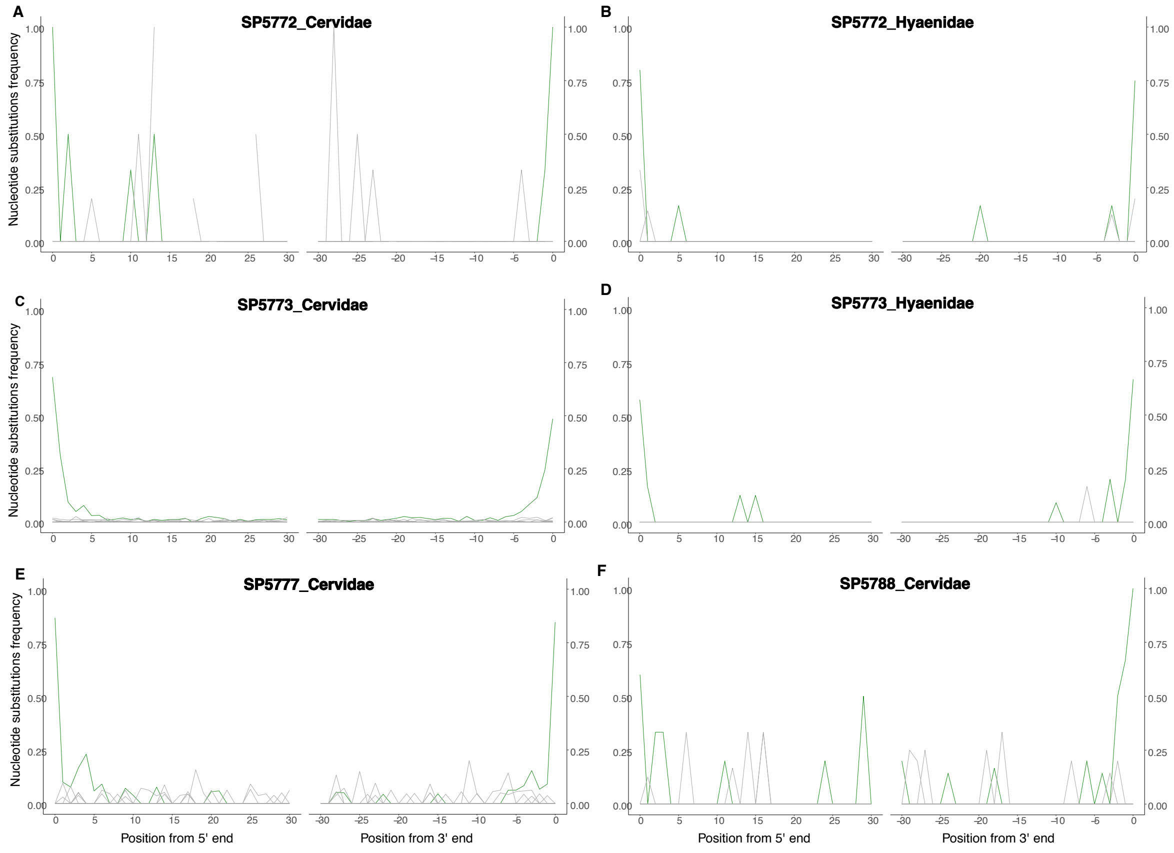

Fig. S1. Authentication of the mtDNA fragments assigned to Cervidae and to Hyaenidae in the Sefunim Cave sediment samples. Green – C to T substitutions; gray – all others. Alignments were made to the *Cervus elaphus* and *Crocuta crocuta* reference mitochondrial genomes, respectively, with the number of fragments noted below reflecting only fragments with a mapping quality of 25 or more that were retained for this analysis. (A) 7 Cervidae fragments in sample SP5772 from layer VII. (B) 25 Hyaenidae fragments in sample SP5772 from layer VII. (C) 965 Cervidae fragments in sample SP5773 from layer V. Note that this panel is identical to the one in Fig 5A. (D) 28 Hyaenidae fragments in sample SP5773 from layer V. (E) 77 Cervidae fragments in sample SP5777 from layer VII (*i.e.*, combining fragments from all six libraries generated from this sample). (F) 13 Cervidae fragments in sample SP5788 from layer VIA. Panels A and F exemplify the difficulty of determining the ancientness of DNA when relatively few fragments from a given taxon are recovered.

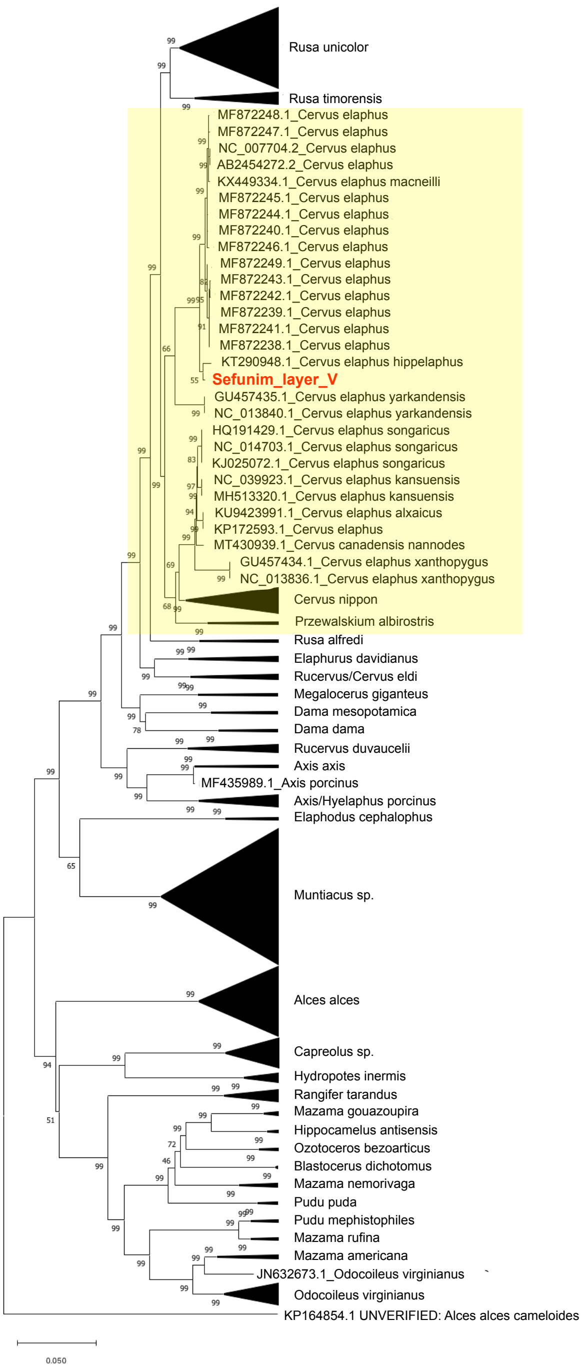

Fig S2. The partial mtDNA genome sequence reconstructed from sample SP5773 from Layer V compared to other Cervidae mitochondrial genomes. The Maximum Likelihood phylogenetic tree is based on the partial mtDNA genome sequence reconstructed from sample SP5773 (shown in red) and 380 previously-sequenced Cervidae mtDNA genomes. Branch lengths are scaled based on the number of substitutions per site, and the support for each branch is based on 500 bootstrap replications. The yellow outline indicates the part of the tree shown in Fig. 6B. The phylogenetic tree was plotted in MEGA X[84] (https://www.megasoftware.net/).

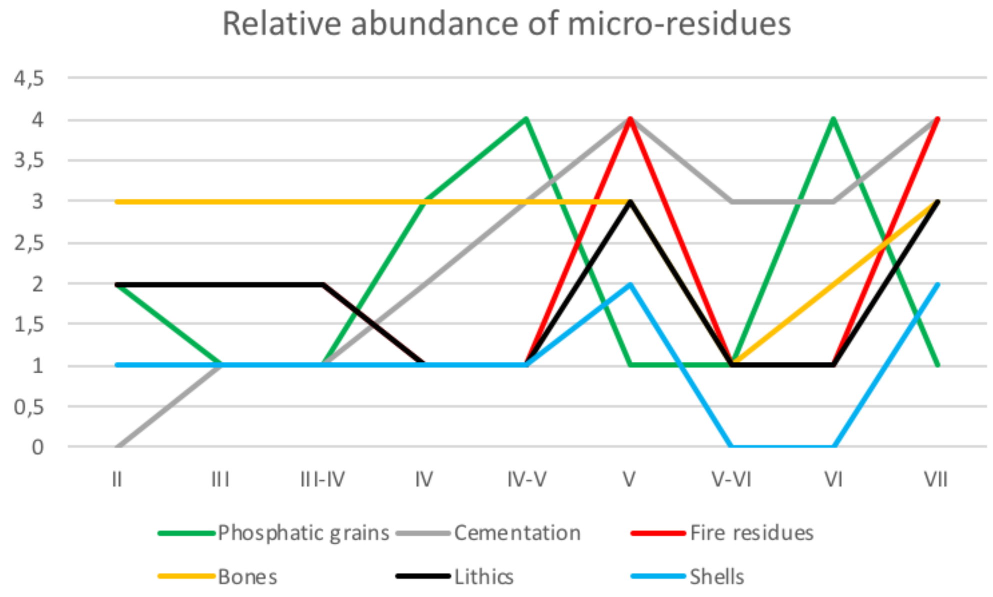

Fig S3. Qualitative assessment of abundance of microscopic residues in thin sections sorted by stratigraphic layers. Yellow - fragments (<2mm) of animal bones; Red - fire residues, including rubified clay aggregates, micro-charcoal, wood ash pseudomorphs and burnt bones; Black – flint fragments; Blue – shell fragments (marine and terrestrial); Gray – precipitation of secondary micritic calcite resulting in cementation of the matrix; Green – phosphatic grains associated with carnivore coprolite fragments.

Supplementary tables

Table S1. List of localities included in Fig. 1, where DNA was successfully retrieved from samples at least 30,000 years old (skeletal remains, sediment samples, or both). Only studies where sequencing data was generated using a DNA library preparation method and where the extent of DNA damage could be evaluated were included.

Locality	Sample type	Reference
60-mile, Yukon Territory, Canada	Skeletal	92
Abri Pataud, France	Sediment	93
Arroyo del Vizcaíno, Uruguay	Skeletal	94
Aufhausener cave, Germany	Skeletal	95
Bacho Kiro, Bulgaria	Skeletal	96
Baishiya, Tibetan plateau	Sediment	97
Bärenfalle BF 24, Austria	Skeletal	95
Baumannshöhle, Rübeland, Germany	Skeletal	98
Belaya Gora, Yakutia, Russia	Skeletal	95
Bolshoy Lyakhovskiy Island, Russia	Skeletal	95
Bolshoy Lyakhovskiy Island, Russia	Skeletal	95
Cave of the Skulls, Israel	Skeletal	17
Certovapec, Slovakia	Skeletal	5
Chagyrskaya, Russia	Both	18,99
Chiquihuite cave, Mexico	Sediment	100
Cioclovina, Romania	Skeletal	101
Denisova, Russia	Both	18,102
Dolganov lake, Russia	Skeletal	95
Dolní Věstonice, Czech Republic	Skeletal	103
Dominion Creek, Dawson City, Yukon, Canada	Skeletal	92
E. Yakutia, Russia	Skeletal	95
Eiros cave, Spain	Skeletal	104
El Sidrón, Spain	Both	18,105
Feldhofer, Germany	Skeletal	105
Forbe's Quarry/Devil's Tower, Gibraltar	Skeletal	106
Fumane, Italy	Skeletal	107
Galería de las Estatuas, Spain	Sediment	19
Geographic Society Cave, Russia	Skeletal	5
Goyet, Belgium	Skeletal	101
Grotte du Renne, France	Skeletal	108
Hohlenstein-Stadel, Germany	Skeletal	109
Hovk-1, Armenia	Skeletal	15
Keremsit River, Indigirka, Tyung, Russia	Skeletal	95
Khomus-Yuryakh Bol. R, Russia	Skeletal	95
Kostenki, Russia	Skeletal	35

Krems, Austria	Skeletal	[101]
Krestovka River, western Beringia	Skeletal	[11]
Kudaro-1, South Ossetia, Southern Caucasus	Skeletal	[15]
Kyttyk Peninsula, Russia	Skeletal	[95]
Les Cottés, France	Both	[18,110]
Medvezhiya cave, Ural Mountains, Russia	Skeletal	[15]
Mezmaiskaya, Caucasus	Skeletal	[105]
Muieri, Romania	Skeletal	[101]
NE Siberia, Russia	Skeletal	[95]
Neumark-Nord, Germany	Skeletal	[111]
Nizhnyaya Tunguska R, Russia	Skeletal	[95]
North Sea, Netherlands	Skeletal	[95]
North Sea, Netherlands	Skeletal	[92]
Ogorokha River, basin of Indigirka River, Russia	Skeletal	[95]
Oimyakon, Yakutia, Russia	Skeletal	[112]
Okladnikov, Russia	Skeletal	[113]
Oskhorkokh, western Beringia	Skeletal	[11]
Paglicci, Italy	Skeletal	[101]
Peştera cu Oase, Romania	Skeletal	[114]
Poolepynten, Svalbard Archipelago, Norway	Skeletal	[115]
Quartz Creek, Yukon Territory, Canada	Skeletal	[95]
Riparo Broion, Italy	Skeletal	[116]
Salkhit, Mongolia	Skeletal	[117]
Scladina, Belgium	Skeletal	[118]
Sher’s site 35, western Beringia	Skeletal	[11]
Siegsdorf, Germany	Skeletal	[95]
Sima de los Huesos, Spain	Skeletal	[12]
Spy, Belgium	Skeletal	[110]
Stajnia, Poland	Skeletal	[119]
Taymir Peninsula, Russia	Skeletal	[120]
Thistle Creek, Yukon, Canada	Skeletal	[121]
Tianyuan, China	Skeletal	[36]
Trou Al'Wesse, Belgium	Sediment	[18]
Ust'-Ishim, Russia	Skeletal	[122]
Vindija, Croatia	Both	[18,123]
Weimar-Ehringsdorf, Germany	Skeletal	[111]
Windischkopf cave, Austria	Skeletal	[104]
Yana, Siberia, Russia	Skeletal	[124]
Zhaodong county, Heilongjiang Province, China	Skeletal	[125]
Zlatý kůň, Czechia	Skeletal	[126]

Table S2. Laboratory IDs and find numbers, elevation and ages of samples dated by radiocarbon.

Lab ID	Square	Find	Layer	Z	Material	14C age BP	St. Dev.	Age cal BP (1σ)	Age cal BP (2σ)	Cal curve
OxA-35137	G49	362	V	5.54	shell	23260	100	26830-26469	26980-26335	Marine20
OxA-31508	H50	618	V	5.89	charcoal	25920	200	30350-29996	30769-29915	IntCal20
OxA-31507	H50	543	V	5.90	charcoal	27880	220	32082-31528	32841-31251	IntCal20
OxA-31632	G50	463	UPPER V	5.90	charcoal	29810	230	34517-34152	34691-33872	IntCal20
OxA-32011	G51	567	V	5.82	charcoal	29900	400	34747-33997	35284-33635	IntCal20
OxA-X-2666-19	H50	1206	V	5.76	charcoal	32220	280	36840-36270	37217-36045	IntCal20
OxA-36779	H49	267	V	5.72	charcoal	32500	240	37074-36484	37455-36290	IntCal20
OxA-X-2666-18	G51	1646	V	5.70	charcoal	32610	280	37300-36551	37770-36231	IntCal20
OxA-37285	I50	1334	V	5.60	charcoal	33400	500	39054-37541	39525-36867	IntCal20
OxA-36781	H50	2103	V	5.59	charcoal	34860	300	40385-39695	40676-39408	IntCal20
OxA-36782	I50	1430	V	5.54	charcoal	33360	280	38719-37580	39151-37233	IntCal20
OxA-36815	H49	727	V	5.40	charcoal	32330	260	36920-36360	37276-36152	IntCal20
OxA-36780	H49	585	VIA	5.29	charcoal	31070	260	35840-35195	36074-34807	IntCal20

Table S3. Laboratory data, dose rates and ages of samples dated by OSL.

Lab name	Layer	$CaCO_3$ [3] (%)	K (%)	U (ppm)	Th (ppm)	Ext. α (µGy/a)	Ext. β (µGy/a)	Ext. γ (µGy/a)	Cosmic (µGy/a)	Dose rate (µGy/a)	OD[1] (%)	N	De[2] (Gy)	Age (ka)
SEF-1	III	6.14	1.35	1.9	3.7	6	1036	585	116	1744±58	15	14/14	36.2±1.5	**20.7±1.1**
SEF-2	III	6.07	1.47	2.8	7.8	11	1280	851	116	2257±79	9	14/14	54.9±1.5	**24.3±1.1**
SEF-3	V	47.8	0.70	1.4	3.0	5	602	384	116	1107±39	14	14/14	45.6±1.9	**41.2±2.2**
SEF-4	V	64.2	0.56	0.37	1.3	2	380	193	116	691±32	13	19/19	45.1±1.4	**65.3±3.7**
SEF-5	VIA	49.6	0.73	0.6	2.0	3	518	280	116	916±41	13	16/16	61.1±2.2	**66.7±3.8**
SEF-6	IV	9.7	1.25	4.6	4.4	13	1279	842	116	2249±79	18	17/19	60.4±1.6	**26.9±1.2**
SEF-7	V	55.8	0.49	2.2	2.8	6	553	404	116	1079±56	22	19/19	49.6±2.5	**50.0±3.3**
SEF-8	VIA	15.0	1.18	2.4	1.8	6	955	525	116	1602±63	18	19/19	81.6±3.7	**51.0±3.1**
SEF-9	VII	28.1	0.75	2.21	1.51	6	687	410	116	1218±44	33	17/19	86.8±3.8	**71.2±4.1**

Notes: Grain size for all samples was 90-125 µm and moisture content estimated at 20±4%. All samples show good recycling ratios within 5% of unity and negligible IR signals. (1) Over-dispersion (OD), a measure of scatter beyond instrumental noise. (2) Calculated using the Central Age Model. (3) For samples SEF-4 to SEF-9, this was measured on the same sediment sample as the dose rate; for samples SEF-1 to SEF-3, the value was taken from the nearest sample taken for soil analyses. Samples SEF-1 to SEF-3 are from [21]. Ext. – External; De – Equivalent dose.

Table S4. Species list (NISP) based on the dental remains.

	Layer III	Layer IV	Layer V	Layer VIA	Layer VII
**Ungulates**					
Equus sp.	1	-	-	-	2
Sus scrofa, wild boar	5	6	3	-	2
Capreolus capreolus, roe deer	2	22	41	-	4
Dama mesopotamica, fallow deer	49	32	102	25	50
Cervus elaphus, red deer	-	-	-	-	1
cf. *Cervus elaphus*	-	-	1	-	1
Medium-large cervid	-	-	2	-	-
Gazella gazella, gazelle	166	83	208	16	16
Capra aegagrus, wild goat	6	3	2	8	16
cf. *Capra aegagrus*	-	-	-	-	2
cf. *Alcelaphus buselaphus,* hartebeest	-	4	2	-	-
Bos primigenius, aurochs	-		1	-	4
Small ungulate	9	6	33	1	2
Medium ungulate	14	12	14	-	3
Large ungulate	1	-	3	-	-
**Carnivores**					
Canis lupus, wolf	3	-	-	-	-
cf. *Canis lupus*	-	-	-	-	1
Vulpes vulpes, red fox	10	2	9	1	-
cf. *Vulpes vulpes*	1	2	4	-	-
Felis sylvestris, wildcat	1	1	8	-	-
Crocuta crocuta, spotted hyena	1	-	1	-	3
Hyaenidae	1	-	-	-	1
Martes foina, marten	-	-	1	-	-
Meles meles, badger	-	-	4	-	-
cf. *Meles meles*	-	-	1	-	-
Small carnivore	3	1	-	-	-
Small to medium carnivore (fox size)	-	-	1	-	-
Medium carnivore (Canis sized)	-	-	1	-	-
**Miscellaneous Small Game**					
Procavia capensis, rock hyrax	-	2	5	-	-
cf. *Lepus*	-	-	1	-	-
**Grand Total**	273	176	448	51	108

NISP – number of identified specimens.

Table S5. Sefunim Cave hyena remains from the current excavation campaign.

Bone	Taxa	Layer	Size (mm)	Measure	Age (years)	Stage
Unciform	*Crocuta*	I-III	24.4/20.4	L/B		
P_4*	*Crocuta*	IIB/IV	23.3/13.9	L/B	1-3	Late juvenile
Scapho-Lunar	*Crocuta*	III	36.8	B		
P^2	Hyaenidae	III				
M_1	*Crocuta*	III	29.8/12.5	L/B	3-6	Early adult
P_4*	*Crocuta*	IIB/IV	23.3/13.9	L/B	1-3	Late juvenile
Navicular	*Crocuta*	IV	29.2/25.9	L/B		
MC5	Hyaenidae	IV	14.5	BD		
Astragal	*Crocuta*	V	40.8	GB		
I1	*Crocuta*	V				
DP_3	*Crocuta*	VII	14.0/6.1	L/B	0-1	Early juvenile
DP_3	Hyaenidae	VII			0-1	Early juvenile
DP_2	*Crocuta*	VII	8.2/4.7	L/B	0-1	Early juvenile
P_2	*Crocuta*	VII	15.9/10.7	L/B		

Abbreviations: M – molar; P – premolar; I – incisor; DP – deciduous premolar; MC – metacarpal; L – Length; B – breadth; BD – breadth distal; GB – greatest breadth. Measurements were made following [127]; age and stage following [128–130].
*Two parts of the same tooth.

Supplementary references

88. Yeshurun, R., Bar-Oz, G. & Weinstein-Evron, M. The role of foxes in the Natufian economy. *Before Farming* **2009**, 1–16 (2009).

89. Rabinovich, R. Man versus carnivores in the Middle- Upper Paleolithic of the Southern Levant. in *Archaeozoology of the Near East V* (eds. Buitenhuis, H., Choyke, A. M., Mashkour, M. & Al-Shiyab, A. H.) 22–39 (ARC-Publicaties, 2002).

90. Stiner, M. C., Arsebük, G. & Howell, F. C. Cave bears and paleolithic artifacts in Yarimburgaz Cave, Turkey: Dissecting a palimpsest. *Geoarchaeology* **11**, 279–327 (1996).

91. Rohland, N. *et al.* The population history of extant and extinct hyenas. *Mol. Biol. Evol.* **22**, 2435–2443 (2005).

92. Paijmans, J. L. A. *et al.* Evolutionary History of Saber-Toothed Cats Based on Ancient Mitogenomics. *Curr. Biol.* **27**, 3330–3336 (2017).

93. Braadbaart, F. *et al.* Heating histories and taphonomy of ancient fireplaces: A multi-proxy case study from the Upper Palaeolithic sequence of Abri Pataud (Les Eyzies-de-Tayac, France). *J. Archaeol. Sci. Reports* **33**, 102468 (2020).

94. Baleka, S. *et al.* Revisiting proboscidean phylogeny and evolution through total evidence and palaeogenetic analyses including Notiomastodon ancient DNA. *iScience* **25**, 103559 (2022).

95. Stanton, D. W. G. *et al.* Early Pleistocene origin and extensive intra-species diversity of the extinct cave lion. *Sci. Rep.* **10**, 12621 (2020).

96. Hublin, J.-J. *et al.* Initial Upper Palaeolithic Homo sapiens from Bacho Kiro Cave, Bulgaria. *Nature* **581**, 299–302 (2020).

97. Zhang, D. *et al.* Denisovan DNA in Late Pleistocene sediments from Baishiya Karst Cave on the Tibetan Plateau. *Science* **370**, 584–587 (2020).

98. Paijmans, J. L. A. *et al.* Historical biogeography of the leopard (Panthera pardus) and its extinct Eurasian populations. *BMC Evol. Biol.* **18**, 156 (2018).

99. Mafessoni, F. *et al.* A high-coverage Neandertal genome from Chagyrskaya cave. *Proc. Natl. Acad. Sci. U. S. A.* **117**, 15132–15136 (2020).

100. Ardelean, C. F. *et al.* Evidence of human occupation in Mexico around the Last Glacial Maximum. *Nature* **584**, 87–92 (2020).

101. Fu, Q. *et al.* The genetic history of Ice Age Europe. *Nature* **534**, 200–205 (2016).

102. Krause, J. *et al.* The complete mitochondrial DNA genome of an unknown hominin from southern Siberia. *Nature* **464**, 894–897 (2010).

103. Fu, Q. *et al.* A Revised Timescale for Human Evolution Based on Ancient Mitochondrial Genomes. *Curr. Biol.* **23**, 553–559 (2013).

104. Barlow, A. *et al.* Partial genomic survival of cave bears in living brown bears. *Nat. Ecol. Evol.* **2**, 1563–1570 (2018).

105. Briggs, A. W. *et al.* Targeted Retrieval and Analysis of Five Neandertal mtDNA Genomes. *Science* **325**, 318–321 (2009).

106. Bokelmann, L. *et al.* A genetic analysis of the Gibraltar Neanderthals. *Proc. Natl. Acad. Sci.* **116**, 15610–15615 (2019).

107. Benazzi, S. *et al.* The makers of the Protoaurignacian and implications for Neandertal extinction. *Science* **348**, 793–796 (2015).

108. Welker, F. *et al.* Palaeoproteomic evidence identifies archaic hominins associated with the Châtelperronian at the Grotte du Renne. *Proc. Natl. Acad. Sci.* **113**, 11162–11167 (2016).

109. Posth, C. *et al.* Deeply divergent archaic mitochondrial genome provides lower time boundary for African gene flow into Neanderthals. *Nat. Commun.* **8**, 1–9 (2017).

110. Hajdinjak, M. *et al.* Reconstructing the genetic history of late Neanderthals. *Nature* **555**, 652–656 (2018).

111. Meyer, M. *et al.* Palaeogenomes of Eurasian straight-tusked elephants challenge the current view of elephant evolution. *Elife* **6**, e25413 (2017).

112. Palkopoulou, E. *et al.* Complete genomes reveal signatures of demographic and genetic declines in the woolly mammoth. *Curr. Biol.* **25**, 1395–1400 (2015).

113. Skoglund, P. *et al.* Separating endogenous ancient DNA from modern day contamination in a Siberian Neandertal. *Proc. Natl. Acad. Sci.* **111**, 2229–2234 (2014).

114. Fu, Q. *et al.* An early modern human from Romania with a recent Neanderthal ancestor. *Nature* **524**, 216–219 (2015).

115. Lindqvist, C. *et al.* Complete mitochondrial genome of a Pleistocene jawbone unveils the origin of polar bear. *Proc. Natl. Acad. Sci.* **107**, 5053–5057 (2010).

116. Romandini, M. *et al.* A late Neanderthal tooth from northeastern Italy. *J. Hum. Evol.* **147**, 102867 (2020).

117. Devièse, T. *et al.* Compound-specific radiocarbon dating and mitochondrial DNA

analysis of the Pleistocene hominin from Salkhit Mongolia. *Nat. Commun.* **10**, 274 (2019).

118. Peyrégne, S. *et al.* Nuclear DNA from two early Neandertals reveals 80,000 years of genetic continuity in Europe. *Sci. Adv.* **5**, 1–10 (2019).

119. Picin, A. *et al.* New perspectives on Neanderthal dispersal and turnover from Stajnia Cave (Poland). *Sci. Rep.* **10**, 14778 (2020).

120. Schubert, M. *et al.* Prehistoric genomes reveal the genetic foundation and cost of horse domestication. *Proc. Natl. Acad. Sci.* **111**, E5661–E5669 (2014).

121. Orlando, L. *et al.* Recalibrating equus evolution using the genome sequence of an early Middle Pleistocene horse. *Nature* **499**, 74–78 (2013).

122. Fu, Q. *et al.* Genome sequence of a 45,000-year-old modern human from western Siberia. *Nature* **514**, 445–449 (2014).

123. Green, R. E. *et al.* A complete Neandertal mitochondrial genome sequence determined by high-throughput sequencing. *Cell* **134**, 416–426 (2008).

124. Sikora, M. *et al.* The population history of northeastern Siberia since the Pleistocene. *Nature* **570**, 182–188 (2019).

125. Yuan, J.-X. *et al.* Molecular identification of late and terminal Pleistocene Equus ovodovi from northeastern China. *PLoS One* **14**, e0216883 (2019).

126. Prüfer, K. *et al.* A genome sequence from a modern human skull over 45,000 years old from Zlatý kůň in Czechia. *Nat. Ecol. Evol.* (2021) doi:10.1038/s41559-021-01443-x.

127. von den Driesch, A. *A Guide to the Measurement of Animal Bones from Archaeological Sites*. (Peabody Museum Press, 1976).

128. Kruuk, H. *The spotted hyena*. (Univ. of Chicago Press, 1972).

129. Stiner, M. C. *Honor among thieves: A Zooarchaeological Study of Neandertal Ecology*. (Princeton University Press, 1994).

130. Diedrich, C. G. Late Pleistocene hyena skeleton remains of a communal/prey depot cave den in the Bohemian Mountains (Czech Republic) – its osteology, taphonomy and palaeoecology. *Acta Zool.* **98**, 66–93 (2017).

Captions for Supplementary Data files

Data S1. List of DNA libraries prepared from the Sefunim Cave samples and from associated negative controls, and the results of testing them for the preservation of ancient DNA. For each biological family identified in the data, the number and percentage of identifiable mtDNA fragments attributed to it are reported, as are the frequencies of cytosine to thymine (C to T) substitutions at the 5' and 3' ends of fragments compared to the reference genome used. Taxa are determined to be of ancient origin if on both extremities, the frequencies are significantly higher than 10%, as determined using a one-sided binomial test (significance level=0.05).

Data S2. Phylogenetic inferences using group-defining informative positions within Cervidae (top) and Hyaenidae (bottom). For each group, the number and percentage of fragments overlapping informative positions and that match the expected base for that group are shown. The number and percentage of fragments that do not match that base, or that present a conflicting result (*i.e.*, when overlapping multiple positions and carrying inconsistent states), are reported as well. The number of informative positions for each group is marked, and we note when these were defined using nearly-fixed (90% frequency or more) rather than fixed differences. Common names for groups are specified when these exist.
